# Comprehensive analysis of the expression patterns and function of the *FTO–LINE1* axis in yak tissues and muscle satellite cells

**DOI:** 10.3389/fvets.2024.1448587

**Published:** 2024-09-05

**Authors:** Zongliang Ma, Zhixin Chai, Huan Yang, Xiangfei Zhang, Hongwen Zhao, Xiaolin Luo, Jincheng Zhong, Zhijuan Wu

**Affiliations:** ^1^Qinghai-Tibetan Plateau Grass-Feeding Livestock Engineering Technology Research Center of Sichuan Province, Southwest Minzu University, Chengdu, China; ^2^Qinghai-Tibetan Plateau Animal Genetic Resource Reservation and Utilization Key Laboratory of Sichuan Province, Southwest Minzu University, Chengdu, China; ^3^Sichuan Academy of Grassland Sciences, Chengdu, China; ^4^Institute of Qinghai-Tibetan Plateau, Southwest Minzu University, Chengdu, China

**Keywords:** *FTO*, *LINE1*, muscle satellite cell, RIP, cell differentiation

## Abstract

**Background:**

The long interspersed nuclear element 1 (*LINE1*) retrotransposon has been identified as a specific substrate for fat mass and obesity-related gene (*FTO*), which facilitates the removal of N^6^-methyladenosine modifications from its targeted RNAs.

**Methods:**

This study examined the dynamic interaction between *FTO* and *LINE1* in yak tissues and muscle satellite cells, utilizing RT-qPCR, RNA immunoprecipitation (RIP), immunofluorescence staining, and techniques involving overexpression and interference of *FTO* and *LINE1* to elucidate the relationship between *FTO* and *LINE1* in yak tissues and muscle satellite cells.

**Results:**

Cloning and analysis of the *FTO* coding sequence in Jiulong yak revealed a conserved protein structure across various *Bos* breeds, with notable homology observed with domestic yak, domestic cattle, and Java bison. Comprehensive examination of *FTO* and *LINE1* gene expression patterns in Jiulong yaks revealed consistent trends across tissues in both sexes. *FTO* mRNA levels were markedly elevated in the heart and kidney, while *LINE1* RNA was predominantly expressed in the heart. Immunoprecipitation confirmed the direct interaction between the FTO protein and *LINE1* RNA in yak tissues and muscle satellite cells. The *FTO*–*LINE1* axis was confirmed by a significant decrease in *LINE1* RNA enrichment following its expression interference in yak muscle satellite cells. Overexpression of *FTO* substantially reduced the expression of recombinant myogenic factor 5 (*MYF5*). However, *FTO* interference had no discernible effect on *MYF5* and myoblast determination protein 1 (*MYOD1*) mRNA levels. Immunofluorescence analysis revealed no alterations in Ki-67 protein expression following *FTO* interference or overexpression. However, phalloidin staining demonstrated enhancement in the myotube fusion rate of yak muscle satellite cells upon *LINE1* interference.

**Conclusion:**

This comprehensive mapping of the *FTO* and *LINE1* mRNA expression patterns establishes a direct interaction between the *FTO* protein and *LINE1* RNA in yak. The findings suggest that *FTO* overexpression promotes muscle satellite cells differentiation, whereas *LINE1* negatively regulates myotube fusion. The study provides fundamental insights into the role of the *FTO*–*LINE1* axis in determining the fate of muscle satellite cells in yak, laying a solid theoretical foundation for future investigations.

## 1 Introduction

The yak (*Bos grunniens*) is endemic to the Qinghai–Tibet Plateau and adjacent regions, serving as a crucial herbivorous livestock species. Its remarkable resilience has made the yak central to the livelihood of local farmers and herdsmen (1). The growth and development of skeletal muscle are pivotal determinants of meat yield and quality in livestock (2). Recent studies have implicated N^6^-methyladenosine (m^6^A) methylation in mammalian skeletal muscle growth (3). m^6^A is the most prevalent form of mRNA methylation in eukaryotes, playing a key role in regulating gene expression (4, 5). This modification process involves several proteins, specifically writer/methyltransferase (6), erasers/demethylase (7), and readers (8), collectively forming a dynamic and reversible regulatory network (9). Among these, the fat mass and obesity-related gene (*FTO*) is a vital m^6^A demethylase that profoundly impacts mammalian development (10). Studies involving chicken myoblasts have demonstrated that *FTO* regulates myogenic differentiation, influencing the expression of myogenic proteins and myosin (11). Despite increasing evidence highlighting the role of *FTO* in regulating skeletal muscle development in other mammals (12), its direct effect on yak muscle satellite cell proliferation and differentiation remains largely unexplored. Consequently, the specific mechanism through which *FTO* regulates the proliferation and differentiation of yak muscle satellite cells remains elusive.

Transposable elements, commonly known as mobile or jumping genes, are DNA segments with the unique ability to insert and excise themselves within the genome, altering their genomic location (13, 14). Among these elements, long interspersed nuclear element 1 (*LINE1*) is a retrotransposon that constitutes a considerable fraction of mammalian DNA, accounting for approximately 18% of mouse DNA, 23% of rat DNA, and 17% of human DNA (15, 16). *LINE1* profoundly impacts mammalian embryonic development, as indicated by the severe disruption of mouse embryonic growth following the deletion of *LINE1* RNA (17). The transient high expression of *LINE1* during zygotic genome activation in early embryonic development of humans and mice suggests that *LINE1* expression is under stringent regulation, playing a crucial role in important physiological processes (18). Recent research indicates that *LINE1* RNA serves as a specific substrate for FTO protein to remove m^6^A modification. In mouse embryos and mouse embryonic stem cells, *FTO* mediates the demethylation of *LINE1* RNA, thereby modulating downstream gene expression by altering *LINE1* RNA levels and local chromatin status (19). However, whether *FTO* protein exerts similar effects on *LINE1* in other tissues and cell types remains unknown.

This study aimed to investigate the interaction between *FTO* and *LINE1* in yak tissues and muscle stem cells and explore whether *FTO* regulates muscle-derived gene expression and myogenic differentiation of yak muscle satellite cells through *LINE1*. The *FTO* coding sequence (CDS) region from the Jiulong yak was cloned, sequenced, and analyzed for bioinformatics analysis. Various tissues, including the heart, liver, spleen, lung, kidney, longissimus dorsi muscle, and pectoral muscle from the Jiulong yak, were used examine the expression of *FTO* and *LINE1*. RIP, immunofluorescence staining, and techniques involving overexpression and interference of *FTO* and *LINE1* were employed to elucidate the relationship between *FTO* and *LINE1* in yak tissues and muscle satellite cells and explore their roles in regulating muscle-derived gene expression and satellite cell differentiation.

## 2 Materials and methods

### 2.1 Animal materials

Jiulong yaks were sourced from Jiulong County in the Ganzi Tibetan Autonomous Prefecture of Sichuan Province. Tissue samples, including the heart, lung, liver, kidney, spleen, longissimus dorsi muscle, and pectoral muscle, were collected from three adult males and three adult females. Immediately after collection, the samples were labeled, submerged in liquid nitrogen, transported to the laboratory, and stored at −80°C. All animal experimental procedures strictly adhered to the guidelines established by the Regional Ethics Committee for Animal Experimentation and complied with the care regulations approved by the Animal Protection and Utilization Committee of Southwest Minzu University.

### 2.2 Isolation and culture of yak muscle satellite cells

The extensor carpi radialis muscles from 5-month-old yaks were surgically isolated under sterile environment. Following disinfection with 75% alcohol, the samples were placed in phosphate-buffered saline (PBS) containing penicillin/streptomycin antibiotics and transported to the laboratory. Blood vessels, fat, and connective tissue were carefully removed, and the muscle tissues were minced finely. The minced samples were resuspended in Dulbecco’s modified Eagle’s medium (DMEM, 11965092; Gibco) mixed with pronase XIV (P5147; Sigma-Aldrich) at a concentration of 1 mg/mL, using a 2:1 v/v ratio of DMEM to pronase XIV solution. This mixture was digested at 37°C for 2 h in a water bath shaker. After digestion, the tissue suspension was allowed to settle for 5 min to collect the supernatant. The supernatant was then filtered through a cell sieve with pore sizes ranging from 70 to 40 μm and centrifuged at 500 × *g* for 10 min to harvest the satellite cells. The isolated cells were resuspended in DMEM supplemented with 20% fetal bovine serum and cultured in a standard culture incubator with humidified air containing 5% CO_2_ at 37°C. The differential adherent method was employed to eliminate myofibroblasts, allowing for the collection and transfer of pure muscle satellite cells to a new Petri dish for further cultivation. Cells reaching 70–80% confluence were either passaged or cryopreserved using trypsin.

### 2.3 *FTO* gene cloning and bioinformatics analysis

Primers were designed using Primer5.0 based on the *FTO* gene sequence of the wild yak (ID: XM_005890937.1), with the following sequences: F: TTAGTAGTGGCGAAGGC, R: ATACCGCCCTT GCCTAA. Yak longissimus dorsi muscle cDNA served as the template for polymerase chain reaction (PCR) amplification. The PCR conditions included an initial denaturation at 95°C for 30 s, followed by cycles of denaturation at 95°C for 10 s, annealing at 60°C for 30 s, and extension at 72°C for 2 min. The amplified PCR product was purified and transformed into *Escherichia coli* DH5-α (TSC-C14; Tsingke). Positive clones were screened by plating the bacterial solution on Luria–Bertani solid medium containing ampicillin and subsequently identified by Tsingke.

Bioinformatics analysis of the cloned CDS sequence of the *FTO* gene from Jiulong yak was conducted using various online tools. ORF finder (NIH) was used to predict open reading frames. ProtParam and ProtScale (Expasy) were utilized to analyze the physicochemical properties of protein. SignalP-4.1 and TMHMM (QIAGEN) were employed to predicting the signal peptide and transmembrane domain, respectively. SOPMA was used to provide insights into the secondary structure, while Swiss-Model was used for tertiary structure prediction. Protein–protein interactions were explored using STRING. Additionally, a phylogenetic tree was constructed using MEGA 11 software.

### 2.4 Reverse transcription quantitative PCR (RT-qPCR) and tissue expression analysis

Total RNA was extracted from yak heart, liver, spleen, lung, kidney, longissimus dorsi, and pectoral muscle tissues using TRIzol reagent (15596018CN; Invitrogen). RNA concentration was determined using a NanoDrop 2000 spectrophotometer (Thermo Fisher Scientific, Beijing), and RNA integrity was assessed via 1.5% agarose gel electrophoresis. The extracted RNA from each tissue served as a template for reverse transcription to cDNA using a reverse transcription kit (639,506; TaKaRa Bio). The cDNA was stored at −20°C until further use.

RT-qPCR primers were designed based on the *FTO* gene sequence of wild yak (ID: XM_005890937.1) and the *LINE1* gene sequence of cattle (ID: DQ000238.1). The primer sequences for *FTO* and *LINE1* were as follows: q*FTO* F: CAGGTGCCAGTCTCGAATTG, R: TGGTTTCCAGAAGCAGACCT; *LINE1* F: GCTGGGAAATCT GGTCAACC, R: TCTGGGAGGTGGGTCATAGA. β-Actin was used as an internal reference, with the primer sequence F: GCAGG TCATCACCATCGG, R: CCGTGTTGGCGTAGAGGT. Additionally, primers for *MYF5* (ID: XM_014480707.1) and *MYOD1* (ID: XM_005896772.1) from wild yak were designed with the following sequences: *MYF5* F: ACGATGGACATGATGGACGG, R: AAACT CGTCCCCGAACTCAC; *MYOD1* F: TCAGACCCTCAGTGC TTTGC, R: CGACAGCAGCTCCATATCCC. qPCR experiments were conducted using TB Green^®^ *Premix EX Taq*^™^ II kit (RR820A, TaKaRa Bio). The reaction conditions included pre-denaturation at 95°C for 30 s, denaturation at 95°C for 10 s, annealing at 60°C for 30 s, and extension at 72°C for 30 s, with a total of 40 cycles. Relative expression levels were calculated using the 2^-ΔΔCt^ method.

### 2.5 RIP

The RIP experiment was performed as described previously (20). Yak heart tissues were lysed using immunoprecipitation (IP) lysate (P0013; Beyotime), and the supernatant was ground on ice. IP experiments were performed using the Dynabeads^™^ Protein A Immunoprecipitation Kit (10006D; Invitrogen) along with an *FTO*-specific polyclonal antibody (27226-1-AP; Proteintech) derived from rabbit. Rabbit immunoglobulin G (IgG) loading control antibody (AC005; ABclonal) served as the negative control. After incubating the antibody and magnetic beads at room temperature, the antigen-containing supernatant was introduced and further incubated at room temperature. Following the removal of the supernatant, the bound antibody was eluted using protease K. After a 30 min incubation, the mixture was placed on a magnetic frame, allowing the supernatant to be transferred to a fresh test tube. RNA was extracted using the TRIzol method and subsequently reverse transcribed into cDNA for qPCR analysis.

### 2.6 Interference with *FTO* and *LINE1* genes

A specific small interfering RNA (siRNA) sequence was designed and synthesized based on the yak *FTO* gene sequence (ID: XM005890937.1). The interference sequences were F: GUGGCA GUGUACAGUUAUATT, R: UAUAACUGUACACUGCCACTT. Yak muscle satellite cells were cultured in 12-well plates. When cell growth reached 70–80% confluence, the medium was replaced with antibiotic- and serum-free DMEM. The si-*FTO* and Lipo8000 transfection reagents (C0533; Beyotime) were combined in a sterile Eppendorf tube and subsequently added to the wells. Six hours post-transfection, the medium was replaced with complete medium, and the cells were incubated for an additional 48 h. The cells were harvested and examined via qPCR. Similarly, a specific siRNA sequence targeting the bovine *LINE1* gene (ID: DQ000238.1) was designed and synthesized. The si-*LINE1* sequences were F: GGACCUAAUUAACCUUAAATT and R: UUUAAGGUUAAUUAGGUCCTT. Yak muscle satellite cells were seeded in 12-well plates. When cell growth was 70–80% confluence, the cells were transfected with si-*LINE1*.

### 2.7 *FTO* gene overexpression

Using the yak *FTO* gene sequence (ID: XM_005890937.1), we designed an overexpression primer sequence incorporating a homologous arm (homologous arm sequences are shown in bold): F: *CTTGGTACCGAGCTCGGATCC*ATGAAGCGGACCCCGACG, R: *TGCTGGATATCTGCAGAATTC*CTAGGGCCTGGTTTCCAGAAG. The target fragment was amplified using cDNA from the longissimus dorsi muscle as a template. The OK Clon ligation kit (AG11807; Agbio) was employed to ligate the amplified fragment into a vector. Positive clones were screened and verified through sequencing. Plasmids were extracted using an endotoxin-free plasmid extraction kit (DP120-01; TIANGEN) and stored at −20°C. Yak muscle satellite cells were cultured in 12-well plates. Once cell growth reached 70–80% confluence, the complete medium was replaced with antibiotic- and serum-free DMEM. Plasmid DNA containing the *FTO* target fragment was combined with Lipo8000 transfection reagent in a sterile Eppendorf tube and introduced into the wells. After 6 h of transfection, the medium was substituted with complete medium, and the cells were incubated for an additional 48 h. The cells were then harvested, and qPCR was performed to assess transfection efficiency and the expression of muscle-derived genes.

### 2.8 Phalloidin staining

Yak muscle satellite cells were seeded in 24-well plates and cultured until they reached 70–80% confluence. At this stage, the complete medium was substituted with differentiation medium consisting of 2% horse serum (16,050,122; GIBCO) and 1% penicillin/streptomycin. The medium was refreshed every 2 days, and cell differentiation was assessed on day 4. Microfilaments within the cells were visualized using Actin-Tracker Red (C2203S; Beyotime) staining. The cells were observed under fluorescence microscopy and images were collected for further analysis.

### 2.9 Immunofluorescence of Ki-67

Yak muscle satellite cells were seeded in 24-well plates and cultured until they reached 70–80% confluence. The cells were then transfected with either pcDNA3.1-*FTO* or si-*FTO*. Following 48 h of incubation, the cells were fixed with 100 μL of 4% paraformaldehyde (BL539A; Biosharp) per ell for 10–20 min. Following fixation, the cells were incubated in PBS containing 0.5% Triton X-100 for 20 min. Blocking was performed with 3% bovine serum albumin at room temperature for 12 h. The cells were then incubated overnight at 4°C in the dark with a diluted Ki-67 rabbit polyclonal primary antibody (PA5-19462; Invitrogen). The secondary antibody was added dropwise and incubated at room temperature for 2 h in the dark. 4′,6-Diamidino-2-phenylindole staining was performed at room temperature for 3–5 min. After a final wash with PBS containing Tween, an anti-quenching agent was applied, and the samples were observed through fluorescence microscope.

### 2.10 Statistical analysis

Statistical analysis was performed using GraphPad Prism 8 software. One-way analysis of variance and multiple comparison tests were performed to determine statistically significance (*p* < 0.05) among the groups. Complete data visualization mapping was also performed using the same software. All experiments were conducted in triplicate.

## 3 Results

### 3.1 Sequence characteristics of the yak *FTO* CDS region

**Figure 1:**
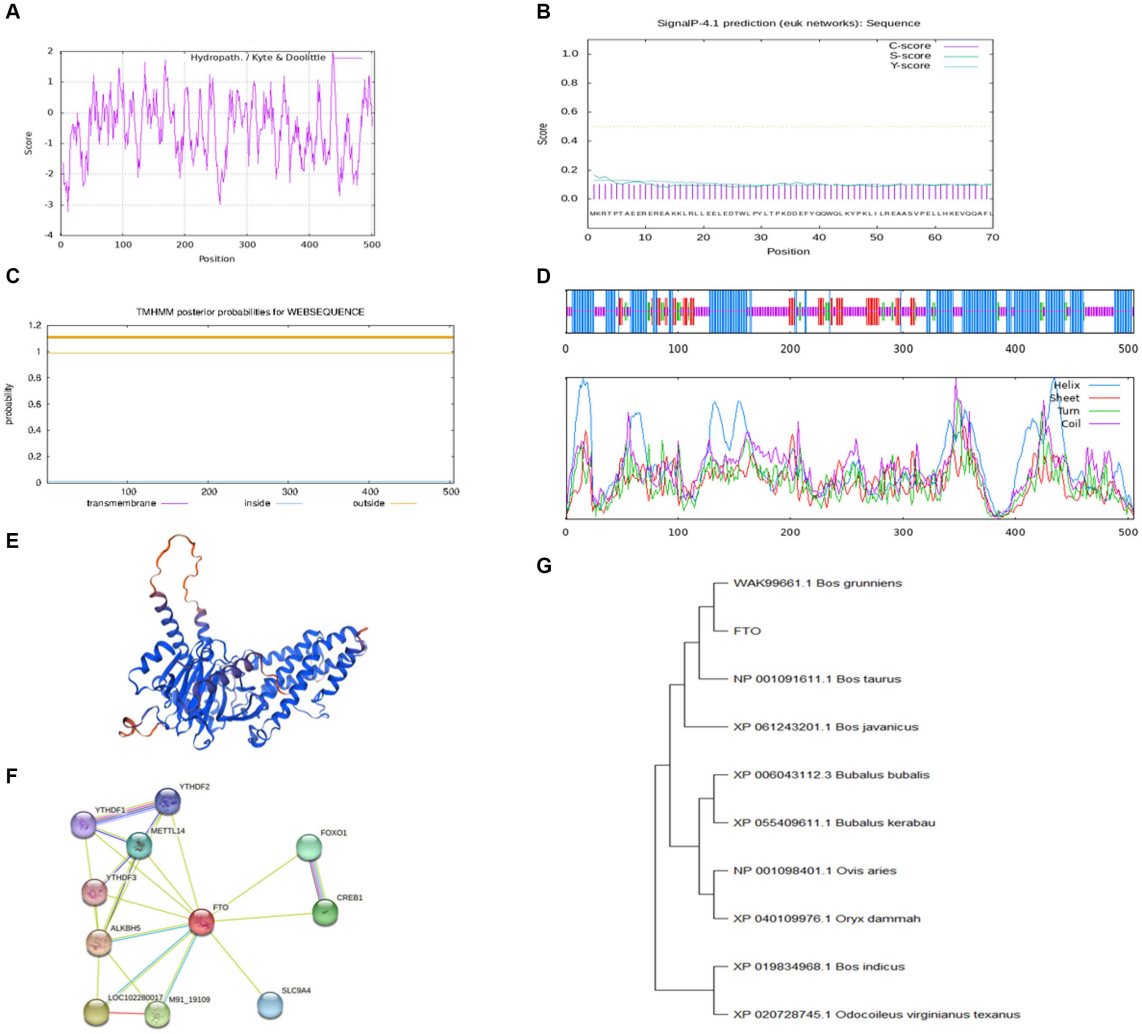
Bioinformatics analysis of *FTO* CDS region in Jiulong yak. **(A)** Hydrophilic/hydrophobic prediction analysis of the protein. **(B)** Signal peptide prediction analysis. **(C)** Transmembrane domain prediction analysis. **(D)** Prediction analysis of protein secondary structure. **(E)** Prediction of protein tertiary structure. **(F)** Prediction analysis of protein interactions. **(G)** The phylogenetic tree of *FTO*.

The CDS region of the *FTO* gene was cloned using cDNA from the longissimus dorsi muscle of Jiulong yak. The cloned sequence, 1,518 bp in length, was assigned GenBank accession number of PP764744. Bioinformatics analysis revealed that the *FTO* protein has a molecular formula of C_2606_H_4047_N_709_O_770_S_27_ and a relative molecular mass of 58495.71 kDa. Leucine was the most prevalent amino acid in the *FTO* protein, comprising 11.3%, while cysteine and histidine were the least abundant at 2.6% each. The *FTO* protein had an instability index of 50.37 and an estimated half-life of 30 h. Additionally, the protein exhibited a fat coefficient of 80.34. Analysis of the hydrophilicity/hydrophobicity of the protein predicted a maximum value of 2 at position 438 and a minimum of −3.22 at position 12, with an overall average hydrophilicity of −0.528, classifying the *FTO* protein as hydrophilic (Figure 1A). SignalP (version 4.1) analysis indicated that the *FTO* protein lacked a signal peptide region, suggesting that it is not a secreted protein (Figure 1B). TMHMM predictions confirmed the absence of a transmembrane structure in the protein (Figure 1C). Secondary structure analysis revealed that α-helix comprised the largest proportion of the *FTO* protein at 43.17%, followed by random coil (38.61%), extended chain (11.49%), and β-turn (6.73%) (Figure 1D). Tertiary structure prediction revealed that the main components of *FTO* protein were still α-helix, extended chain, and random coil (Figure 1E).

Analysis conducted using STRING software identified a strong correlation between yak *FTO* and several other genes, including AlkB homolog 5, cAMP responsive element binding protein 1, methyltransferase-like 14, forkhead box protein O1, and **YTH domain family member 1** (Figure 1F). Furthermore, BLAST nucleotide sequence alignment revealed remarkable similarity (up to 99.74%) between the *FTO* gene of Jiulong yak and its counterparts in wild yak (ID: XM_005890937.1) and domestic yak (ID: OM640141.1). Significant similarities were also noted with hybrids of North American wild yak (ID: XM_010861935.1; 99.41%), domestic cattle (ID: NM_001098142.1; 99.41%), and zebu and yellow cattle (99.34%). The *FTO* gene sequences of Jiulong yak were compared with those of nine distinct species retrieved from GenBank using a phylogenetic tree constructed by the MEGA (version 11) software. The results indicated the highest homology between Jiulong yak and *B. grunniens*, followed by *Bos taurus* and *Bos javanicus* (Figure 1G).

### 3.2 Expression analysis of *FTO* and *LINE1* in yak tissues

**Figure 2:**
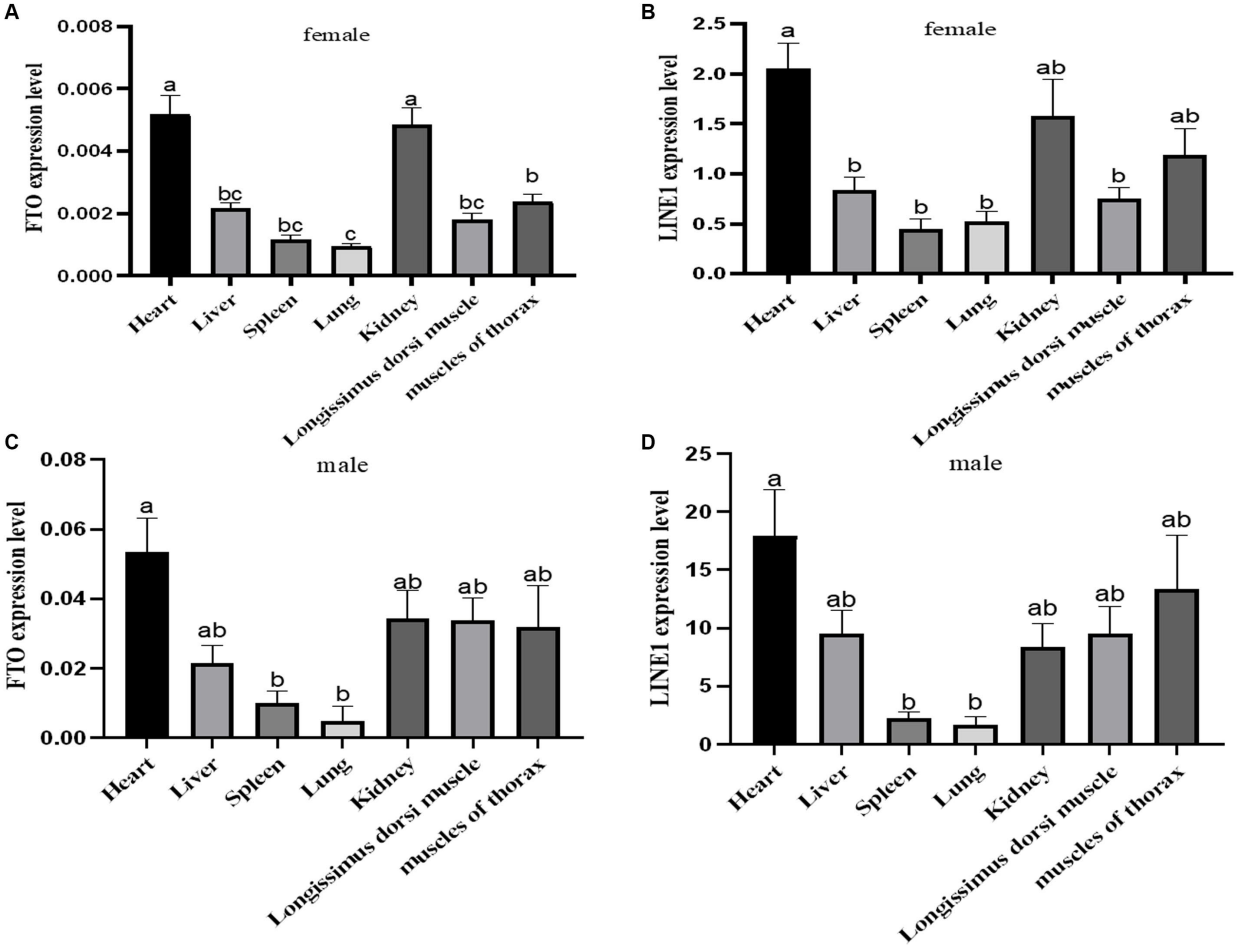
Expression analysis of *FTO* and *LINE1* in yaks. **(A)** Analysis of *FTO* expression in female yak tissues. **(B)** Analysis of *LINE1* expression in female yak tissues. **(C)** Analysis of *FTO* expression in male yak tissues. **(D)** Analysis of *LINE1* expression in male yak tissues.

The expression levels of *FTO* and *LINE1* were evaluated in the heart, liver, spleen, lung, kidney, longissimus dorsi, and pectoral muscle tissues of adult male and female Jiulong yaks. Both *FTO* and *LINE1* exhibited consistent expression patterns across these tissues, with no observable sex-specific differences (Figure 2). In female yak tissues, *FTO* expression was notably higher in the heart and kidney tissues than in the other tissues, while *LINE1* expression was predominantly elevated in the heart. Similarly, in male yak tissues, the heart tissue demonstrated significantly increased expression levels of both *FTO* and *LINE1* compared to the spleen and lung tissues.

### 3.3 Interaction between *FTO* and *LINE1* in yak tissues

**Figure 3:**
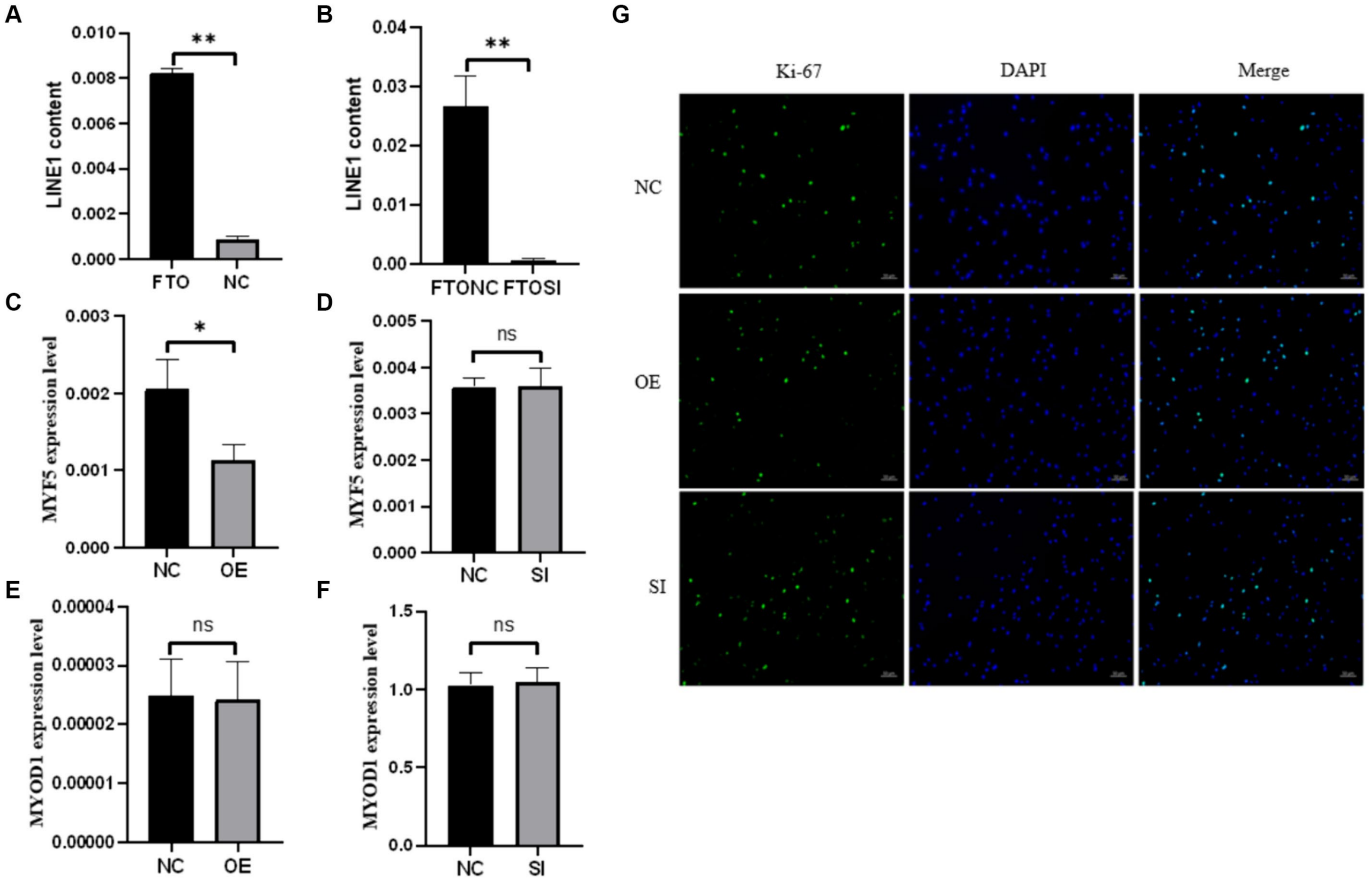
The RIP experiment of *FTO* and the effects of *FTO* modulation on the proliferation and differentiation of muscle satellite cells. **(A)** RIP assay in heart tissue. **(B)** RNA immunoprecipitation analysis using the *FTO* antibody after *LINE1* interference. Impact of *FTO* modulation on the expression of *LINE1* and muscle-specific genes in muscle satellite cells. **(C)** Analysis of *MFY5* expression upon *FTO* overexpression. **(D)** Assessment of *MYF5* expression after *FTO* interference. **(E)** *MYOD1* expression analysis following *FTO* overexpression. **(F)** *MYOD1* expression profiling after *FTO* interference. **(G)** Immunofluorescence-based detection of Ki-67.

To explore the interaction between *FTO* protein and *LINE1* RNA in yak tissues, RIP using an *FTO* antibody was performed on yak heart tissues, followed by qPCR analysis of *LINE1* expression, with IgG as a negative control. Our results indicated a direct interaction between *FTO* protein and *LINE1* RNA specifically in yak heart tissue (Figure 3A). To further validate this interaction, *LINE1* expression was knocked down in yak muscle satellite cells by transfecting them with si-*LINE1* and repeating the RIP assay. A significant decrease in *LINE1* expression was evident after si-*LINE1* transfection, accompanied by a reduction in the binding affinity of *FTO* to *LINE1* (Figure 3B).

Upon modulating *FTO* levels in muscle satellite cells, *MYF5* expression remained unaffected by *FTO* interference (Figure 3C), whereas *FTO* overexpression significantly downregulated *MYF5* expression (Figure 3D). However, *MYOD1* expression was not influenced by *FTO* knockdown (Figure 3E) or overexpression (Figure 3F). Additionally, cell immunofluorescence results indicated that Ki-67 expression was not significantly affected by changes in *FTO* levels (Figure 3G). These results suggest that altering *FTO* expression does not affect the proliferation of yak skeletal muscle satellite cells; however, *FTO* overexpression tends to promote the myogenic differentiation of these cells.

### 3.4 Impact of *LINE1* downregulation on myotube formation in yak muscle satellite cells

**Figure 4:**
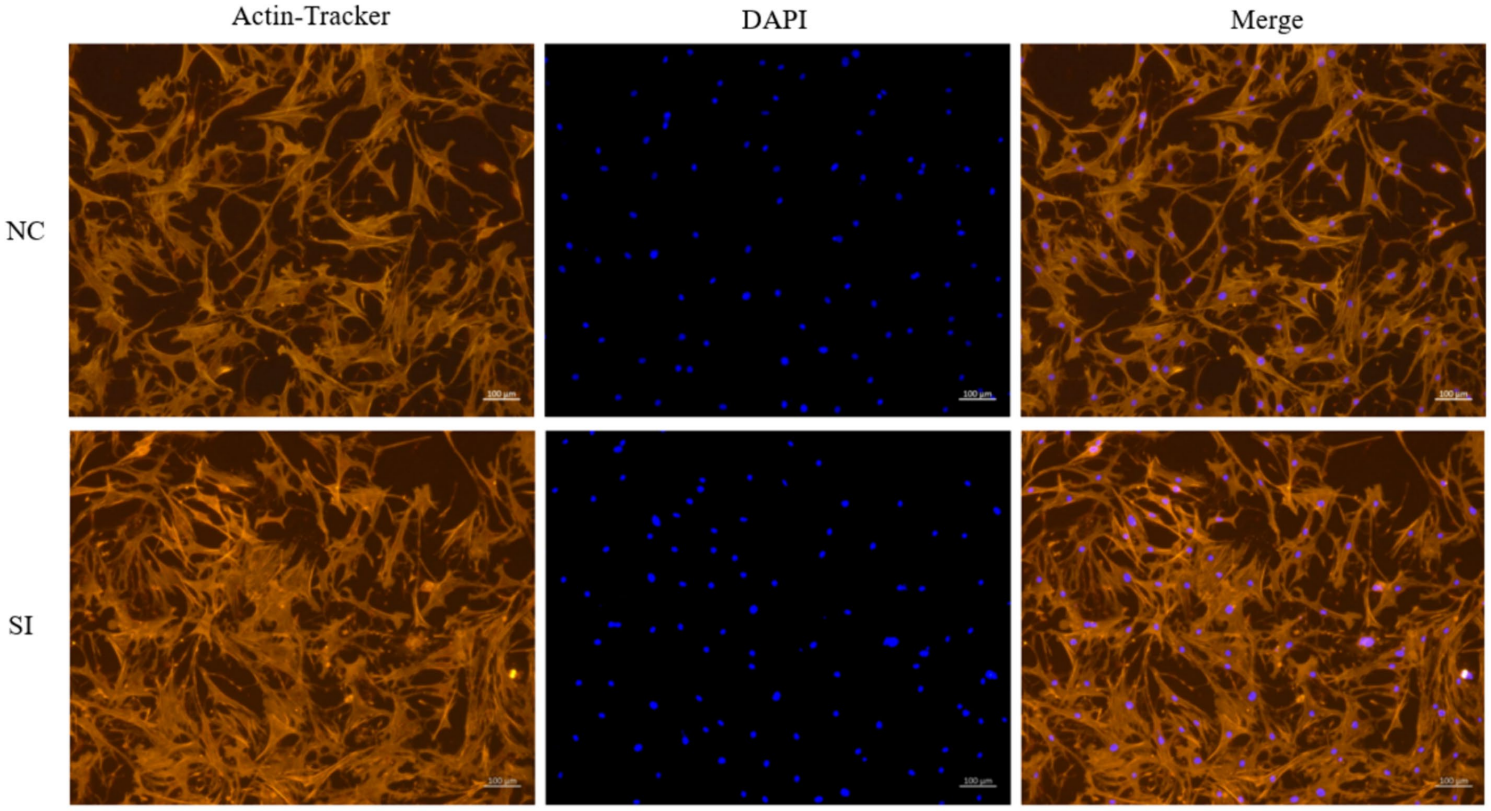
Examination of muscle tube fusion via AcN-tracker Red staining after LINE1 interference. Yak muscle satellite cells were cultured in differentiation medium for four days, and the formation of myotubes was detected by phalloidin staining, and the nucleus were stained with DAPI.

Following siRNA-induced downregulation of *LINE1* expression in yak muscle satellite cells, the differentiation process was visualized on day 5 through phalloidin staining. Comparative analysis between the si-*LINE1* and si-NC groups revealed a significant enhancement in the myotube fusion rate upon *LINE1* downregulation (Figure 4).

## 4 Discussion

*FTO* plays a pivotal role as an m^6^A demethylase in mammalian growth and development (10), with *LINE1* recently identified as a key substrate, enabling *FTO* to eliminate m^6^A modifications from the target RNA (19). During mouse embryonic development, *FTO* regulates the m^6^A modification of chromatin-related RNA, particularly *LINE1* RNA, transcribed from transposon elements in mouse embryonic stem cells (19). This modification of *FTO* shapes the local chromatin state, thereby affecting the opening and closing of genes containing *LINE1* elements. This study aimed to elucidate the interaction between *FTO* and *LINE1* in yak tissues and muscle satellite cells to determine the regulatory mechanism within the *FTO*–*LINE1* axis in muscle development.

In this study, we successfully cloned the 1,518 bp CDS region of the *FTO* gene from the Jiulong yak, encoding 505 amino acids. Our bioinformatics analysis indicated that the secondary structure of *FTO* protein predominantly consists of α-helices, with no transmembrane domains or signal peptides, suggesting that *FTO* is neither a transmembrane nor a secretory protein, aligning with previous observations in goat (21) and pig *FTO* (22). This cross-species consistency underscores the remarkable conservation of the *FTO* gene. Tissue-specific expression analysis of *FTO* and *LINE1* in adult yaks revealed distinct patterns. In female yaks, *FTO* expression was significantly higher in the heart and kidney than in the other tissues. In male yaks, heart tissue exhibited predominantly higher *FTO* expression followed by the spleen and lung. These findings diverge from those of a previous study on 18-month-old Datong yaks, where heart tissue exhibited peak *FTO* expression, exceeding the expressions in the liver, spleen, lung, kidney, and longissimus dorsi (23). Comparable patterns of elevated *FTO* expression were observed in the liver and subcutaneous fat of 3-year-old Guangling donkeys (24). Varying *FTO* expression patterns, with high expression in the liver, have been reported in Tan sheep (25) and the hypothalamus and liver of Qianbei Brown goat (26). Our study identified consistent trends in *FTO* expression across tissues in both male and female yaks, although the actual expression levels differed. Our findings contribute to the broader understanding of *FTO* expression patterns and regulatory mechanisms, offering valuable insights into its potential functions in various biological processes.

Transposable elements constitute a significant portion of many eukaryotic genomes, with nearly half of the human genome consisting of transposons (14). *LINE1,* as a retrotransposon, plays a pivotal role in facilitating chromatin opening within cells (27). Jachowicz et al. (28) demonstrated that premature silencing of the *LINE1* element in mouse embryonic cells reduces chromatin accessibility, while its prolonged activation helps counteract natural chromatin compression during developmental processes. This *LINE1* activation influences global chromatin accessibility in early developmental stages, suggesting that retrotransposon activation is essential for developmental progression. We observed, both *FTO* and *LINE1* were widely expressed across various tissues in both male and female yaks. *LINE1* expression was significantly higher in the heart, kidney, and chest muscle of female yaks than in the other four tissues examined. Similarly, in male yaks, *LINE1* expression was markedly increased in the heart compared to that in the spleen and lung. Consistent expression patterns of *FTO* and *LINE1* across diverse tissues, independent of sex, suggest a possible interaction between these molecules in yak tissues.

Although a direct relationship between *FTO* and *LINE1* remains limited, indirect evidence suggests a significant connection between them. *FTO* has been associated with skeletal muscle development and aging. In older individuals, *FTO* mRNA expression in the skeletal muscle is reduced by approximately 28% compared to that in younger individuals (29), a pattern also observed in aging Tibetan sheep (30). Exercise has been shown to attenuate the impact of *FTO* gene expression in skeletal muscle (31). Similarly, *LINE1* expression correlates with skeletal muscle development and aging. For instance, the expression of *LINE1* mRNA in the skeletal muscle of 24- and 36-month-old mice exceeds that of 5-month-old mice (32). In mice, rats, and humans, *LINE1* expression increases with age, while prolonged exercise decreases its expression in human skeletal muscle (33). These findings suggests a potential correlation between *FTO* and *LINE1* expression changes during skeletal muscle development and aging. Therefore, investigating the interplay between the demethylase *FTO* and its substrate *LINE1* holds significant promise in elucidating the molecular mechanism underlying skeletal muscle development and aging.

To investigate the potential interaction between *FTO* and *LINE1* in yak tissues, we performed RIP analysis on heart tissue, where both *FTO* and *LINE1* expression were highest. Our results demonstrated a direct interaction between *FTO* protein and *LINE1* RNA, which was further validated in yak muscle satellite cells. A significant decrease in the binding of *FTO* protein to *LINE1* RNA was observed by performing RIP experiments following siRNA-mediated interference of *LINE1* expression in these cells, further confirming the direct interaction between the two molecules.

During aging, individuals experience muscle mass loss, increased chronic inflammation, and cellular senescence (34), factors that may influence *FTO* gene expression. In Tibetan sheep, aging is accompanied by alterations in muscle fiber composition from slow to fast fibers, along with changes in energy metabolism (30). The elevated expression of *LINE1* in older mice, rats, and humans relative to their younger counterparts is potentially attributable to age-related modifications in chromatin status, augmenting to higher transcriptional activity of *LINE1* (33). *FTO* contributes to the preservation of slow muscle fibers in mice, both *in vivo* and *in vitro*, through its demethylation activity (12). These results suggest a correlation between the trends of *FTO* and *LINE1* expression changes during skeletal muscle development and aging.

*FTO* also plays a crucial role in myogenic differentiation (35). *MYF5* and *MYOD1* are essential myogenic regulatory factors that regulate muscle development and regeneration by directing the activation and differentiation of muscle precursor cells into mature muscle fibers (36). During muscle stem cell differentiation, *MYF5* expression decreases (37), while *MYOD1* initiates the transcription of skeletal muscle-specific genes and induces cell cycle arrest, essential for muscle cell differentiation and initiating myogenesis (38). *FTO* knockdown enhances bovine myoblast proliferation while inhibiting differentiation, whereas its overexpression stimulates myoblast differentiation (39). In our study, we observed a significant decrease in *MYF5* expression upon *FTO* overexpression, while *MYOD1* expression remained unchanged, suggesting the involvement of compensatory regulatory mechanisms. Immunofluorescence analysis of Ki-67 revealed no significant changes following *FTO* expression modification, suggesting that *FTO* does not significantly influence the proliferation of yak muscle satellite cells. This finding contradicts previous studies where *FTO* knockdown increased bovine muscle stem cell proliferation (39) and decreased *Ki-67* expression in mouse tumor cells (40). Further examination is needed to elucidate the role and mechanism of *FTO* in regulating cell proliferation.

In this study, we further manipulated *LINE1* expression, the primary substrate for *FTO*-mediated m^6^A demethylation, using siRNA. Phalloidin staining revealed a significant increase in myotube fusion following *LINE1* downregulation. This myotube fusion process is intricately linked to chromatin dynamics, particularly nuclear mechanics regulation and chromatin accessibility (41). Disruption of actomyosin complexes can alter nuclear morphology and decrease chromatin accessibility, which is crucial for myogenic differentiation (42), as it enables regulatory proteins, such as transcription factors, to access genes and activating the expression of muscle-specific genes. Interfering with microtubules in mouse embryonic fibroblasts can partially restore nuclear morphology and chromatin accessibility without compromising cellular force generation (43). However, in certain contexts, low chromatin accessibility is essential to maintain cellular function stability. The observed decrease in *LINE1* mRNA expression may positively regulate chromatin openness, promoting myogenic differentiation and myotube fusion in yak muscle satellite cells. This function of *LINE1* appears to be dependent on the m^6^A demethylase activity of *FTO*, given the direct interaction between *FTO* protein and *LINE1* RNA.

## 5 Conclusion

The cloning and analysis of the Jiulong yak *FTO* gene CDS sequence revealed its conservation across species. Tissue analysis showed similar expression patterns of *FTO* and *LINE1* RNA across yak tissues, with a direct *FTO*–*LINE1* RNA interaction confirmed experimentally. The overexpression of *FTO* downregulated *MYF5* mRNA expression in yak muscle cells without affecting *MYOD1* expression or cell proliferation, while disrupting *LINE1* expression enhanced muscle cell fusion. These findings provide valuable insights into the role of the *FTO*–*LINE1* axis in yak muscle development.

## Data Availability

The datasets presented in this study can be found in online repositories. The names of the repository/repositories and accession number(s) can be found at: https://www.ncbi.nlm.nih.gov/, PP764744.
